# Diapocynin, a Dimer of the NADPH Oxidase Inhibitor Apocynin, Reduces ROS Production and Prevents Force Loss in Eccentrically Contracting Dystrophic Muscle

**DOI:** 10.1371/journal.pone.0110708

**Published:** 2014-10-17

**Authors:** Hesham M. Ismail, Leonardo Scapozza, Urs T. Ruegg, Olivier M. Dorchies

**Affiliations:** School of Pharmaceutical Sciences, University of Geneva and University of Lausanne, Geneva, Switzerland; University of Minnesota, United States of America

## Abstract

Elevation of intracellular Ca^2+^, excessive ROS production and increased phospholipase A_2_ activity contribute to the pathology in dystrophin-deficient muscle. Moreover, Ca^2+^, ROS and phospholipase A_2_, in particular iPLA_2_, are thought to potentiate each other in positive feedback loops. NADPH oxidases (NOX) have been considered as a major source of ROS in muscle and have been reported to be overexpressed in muscles of mdx mice. We report here on our investigations regarding the effect of diapocynin, a dimer of the commonly used NOX inhibitor apocynin, on the activity of iPLA_2_, Ca^2+^ handling and ROS generation in dystrophic myotubes. We also examined the effects of diapocynin on force production and recovery ability of isolated EDL muscles exposed to eccentric contractions *in vitro*, a damaging procedure to which dystrophic muscle is extremely sensitive. In dystrophic myotubes, diapocynin inhibited ROS production, abolished iPLA_2_ activity and reduced Ca^2+^ influx through stretch-activated and store-operated channels, two major pathways responsible for excessive Ca^2+^ entry in dystrophic muscle. Diapocynin also prevented force loss induced by eccentric contractions of mdx muscle close to the value of wild-type muscle and reduced membrane damage as seen by Procion orange dye uptake. These findings support the central role played by NOX-ROS in the pathogenic cascade leading to muscular dystrophy and suggest diapocynin as an effective NOX inhibitor that might be helpful for future therapeutic approaches.

## Introduction

Duchenne muscular dystrophy (DMD) is a very severe muscle disease characterized by progressive skeletal muscle wasting. It is provoked by mutations in the gene encoding the protein dystrophin, leading to its absence in skeletal muscles of DMD patients [1], causing loss of the dystrophin-glycoprotein complex and improper mechano-transduction. Dystrophin-deficient myofibers are more susceptible to contraction-induced injury, leading to necrosis, muscle wasting and premature death [2].

There are numerous consequences of the absence of dystrophin on cellular signalling affecting muscle function and homeostasis of the myofiber. Of primary concern is the upregulated influx of Ca^2+^ through channels and transient breaks in the membrane [3]. Indeed, a number of studies have reported chronic elevation in intracellular Ca^2+^ concentrations in skeletal muscle fibers or in cultured myotubes from DMD patients and mdx mice, a mouse model for DMD. Stretch-activated channels (SACs) and store-operated channels (SOCs) are considered as candidates for mediating such an influx [4]. Another consequence of the lack of dystrophin is increased activity of the calcium-independent isoform of phospholipase A_2_ (iPLA_2_), observed in biopsies from DMD patients [5] and mdx mice [6]. This enzyme has been reported to activate SOCs and SACs as evidenced by iPLA_2_ inhibition [7].

Another downstream consequence of the lack of dystrophin is increased reactive oxygen species (ROS) production. Markers of oxidative stress and lipid peroxidation are elevated in dystrophic muscles, even before the first symptoms of the disease appear (reviewed in Tidball and Wehling-Henricks [8]). Furthermore, ROS have been proposed as possible mediators of dystrophic muscle damage as they can activate several Ca^2+^ channels and promote lipid peroxidation, resulting in sarcolemmal fragility and subsequent Ca^2+^ influx through micro-ruptures, seen in dystrophic muscle [3]. In fact, reciprocal amplification of Ca^2+^ influx and ROS production results in a vicious cycle that appears to be central in the dystrophic pathology [9], [10]. Several studies over the past decade were conducted in mdx mice to evaluate the effectiveness of anti-oxidants in ameliorating the pathological process, all of which showed benefit on selected parameters [10]–[14]. On the other hand, clinical trials conducted with anti-oxidants did not show an improvement and some even resulted in deterioration of the condition, which was attributed to lack of selectivity of the chosen anti-oxidant interventions against a defined target [15].

For a long time, mitochondria have been considered the main source of ROS in skeletal muscle during exercise. NADPH oxidases (NOXes), lipoxygenases, monoamine oxidase and xanthine oxidase have been proposed as other relevant sources of ROS in muscle cells (reviewed in [16]). It was recently shown that NOXes contribute to ROS production in skeletal muscle to a larger extent than mitochondria [9], [17], [18], which makes NOXes attractive targets to treat DMD.

The NOX family members are transmembrane proteins that transport electrons across biological membranes to reduce oxygen to superoxide or H_2_O_2_ [19]. Total mRNA from skeletal muscle contains NOX4 and NOX2. NOX4 is a constitutively active monomeric enzyme, whereas NOX2 requires the translocation of several regulatory subunits (p22^ph^°^x^, p47^ph^°^x^ and p67^ph^°^x^) to the membrane-spanning subunit gp91^ph^°^x^ to be active [19]. NOX2 and all of its subunits, except p22, are overexpressed in skeletal muscles from 19-day old mdx mice, just before the onset of necrosis, suggesting an early involvement of NOX in the pathology seen in DMD [20]. Another study showed that NOX4 mRNA is increased 5-fold in the left ventricles from 9–10 months old mdx mice [21].

In view of the importance of NOXes in various pathologies, a search for potent, efficacious, selective and non-toxic NOX inhibitors has been started. Several classes of compounds such as pyrazolopyridine, pyrazolopyrimidine, triazolopyrimidine, tetrahydroindole, and fulvalene analogues have been shown to inhibit NOX activity (for a review see Kim *et al.* [22]), and the synthetic peptide gp91ds-*tat* has also been shown to potently inhibit NOX2 [23]. However, the most commonly used experimental NOX inhibitor to date is apocynin. Apocynin was found to inhibit ROS production by NOXes in phagocytic cells, whereas it failed to do so and even promoted ROS production in non-phagocytic cells [24]. One explanation for this discrepancy is that phagocytic cells efficiently convert inactive apocynin monomers into active diapocynin through a peroxidase-mediated dimerization that is not operating in other cell types [24]–[26].

In the current study, we synthetized diapocynin and evaluated its effect on key mediators in the pathogenesis of DMD, namely ROS production, iPLA_2_ activity and Ca^2+^ influx through SOC and SAC in dystrophic skeletal muscle cells. We also investigated its effect on force loss induced by eccentric contractions of isolated dystrophic fast twitch muscles. Not only did diapocynin inhibit ROS production in dystrophic myotubes, but also iPLA_2_ activity and Ca^2+^ influx. In addition, it reduced force loss induced by eccentric contractions to near-control values.

## Materials and Methods

### Pharmacological treatments

The present investigations used a combination of pharmacological, cell biological and functional assays. In preliminary experiments, diapocynin showed significant alterations of the readouts at concentrations of 100 and 300 µM and were selected for further evaluations. For comparative purposes, apocynin was tested at a concentration 300 µM. The other compounds (BEL, BTP2, colchicine, DPI, GsMTx-4, streptomycin) were used at concentrations commonly reported in previous investigations in the field. These concentrations are around 3–10 times their IC_50_ at the targets in order to ensure maximal inhibitory effects [20], [27]–[29].

### Diapocynin synthesis and characterization

Diapocynin was synthetized from apocynin (Sigma, Buchs, Switzerland) through an oxidative coupling reaction in the presence of ferrous sulfate and sodium persulfate as described [30]. The brown precipitate formed after this reaction was dissolved in 3N ammonia, re-crystallized in 6N HCl and washed 3 times with boiling water to yield pure diapocynin, as verified by NMR and mass spectrometry.

### Cell culture

Myotubes were prepared from EDL-MDX-2 myoblasts co-cultured on a feeder layer of 10T½ fibroblasts as described previously [29], [31]. Briefly, EDL-MDX-2 and 10T½ were propagated on collagen-treated and on uncoated Petri dishes (Falcon, Becton Dickinson), respectively, in high-mitogen containing proliferation media. Cells were detached with trypsin and suspensions containing 80,000 EDL-MDX-2 myoblasts and 60,000 mitomycin C-inactivated 10T½ fibroblasts per ml were seeded in 24-well plates coated with 1 µg/cm^2^ Matrigel (Becton Dickinson), 0.5 ml per well. After 2 days, myotube formation was induced by changing the proliferation medium to a low-mitogen containing differentiation medium. After 3–4 days contracting myotubes were obtained.

### Determination of ROS production

ROS production was measured using 2′,7′-dichlorohydrofluorescein-diacetate (DCFH-DA, Invitrogen, Zug, Switzerland), a probe that readily enters cells, which, upon de-acetylation by cellular esterases reacts with a variety of reactive oxygen/nitrogen species to yield fluorescent 2′,7′-dichlorofluorescein (DCF). To perform these experiments, myotube cultures were washed twice with Ca^2+^-free physiological salt solution (PSS−; composition in mM: HEPES 5, KCl 5, MgCl_2_ 1, NaCl 145, glucose 10, EGTA 0.2) and incubated with 20 µM of DCFH-DA for 1 h to allow sufficient loading of the cells. Subsequently, compounds to be tested were added and the development of the fluorescent signal was monitored with a FLUOStar Galaxy fluorimeter (BMG Laboratories, Offenburg, Germany) as described [6].

### Determination of PLA_2_ activity

PLA_2_ activity was measured using the probe PED-6 (Invitrogen), which is cleaved by PLA_2_ to release BODIPY, a green fluorescent compound. Briefly, EDL-MDX-2 myotube cultures were washed twice with PSS− and incubated with test compounds for 20 min. Subsequently, PED-6 (1 µM) was added and the fluorescence increment was measured over a period of 30 min at 37°C as described [32].

### ^45^Ca^2+^ influx triggered by store depletion and hypo-osmotic shock

^45^Ca^2+^uptake was quantified as described by Ismail et al. [29]. To measure the activity of SACs, myotube cultures were washed twice with PSS containing 1.2 mM Ca^2+^ (PSS+), pre-incubated at 37°C for 15 min with test compounds and then exposed for 5 min to 200 µl/well of a hypo-osmotic PSS+ (100 mOsm obtained by decreasing the NaCl concentration from 145 to 25 mM) containing 1 µCi/ml of ^45^Ca^2+^. Plates were then placed on ice, and cultures were washed 4 times with ice-cold PSS− to remove remaining extracellular ^45^Ca^2+^ before being lysed with 0.5 ml of 1N NaOH. The radioactivity in the lysates was determined by scintillation counting (Ultima Gold, Packard, Groningen, NL) using a beta-counter (LKB Wallac 1217 Rackbeta, Turku, Finland).

To study the activity of SOCs, the cultures were washed twice with PSS+, pre-incubated for 15 min at 37°C with test compounds in 200 µl Ca^2+^-free PSS, and further exposed to 5 µM thapsigargin to deplete intracellular Ca^2+^ stores, in the presence of test compounds. After 10 min, PSS+ containing 1 µCi/ml ^45^Ca^2+^ was added and uptake was measured after another 10 min. ^45^Ca^2+^ was quantified as above.

### Isolated muscle experiments

To evaluate whether diapocynin modulates force loss in eccentrically contracting muscles, a method described earlier [29] was used. Dystrophic (mdx^5Cv^) and wild type (C57BL/6J) mice were maintained in the animal facility of the Geneva-Lausanne School of Pharmaceutical Sciences and used in compliance with the local rules on animal experimentation and welfare (Authorization #106/3626/0 delivered by the Cantonal Veterinary Office of Geneva and approved by the Swiss Veterinary Office). Mice between 8 and 12 weeks of age were anesthetized, the *extensor digitorum longus* (EDL) muscles were exposed, and their proximal and distal tendons were tied with silk sutures. Then, EDL muscles were excised and transferred to a 10 ml horizontal chamber of a muscle-testing device designed for delivering eccentric contractions (model 305C-LR, Aurora Scientific Inc., Ontario, Canada). The muscle chamber was filled with a physiological Ringer solution (composition in mM: NaCl 137, NaHCO_3_ 24, glucose 11, KCl 5, CaCl_2_ 2, MgSO_4_ 1, NaH_2_PO_4_ 1, pH 7.4) that contained 25 µM D-tubocurarine and was continuously bubbled with 95% O_2–_5% CO_2_. Muscles were stimulated by 0.2 ms square wave pulses generated by a Grass S88X stimulator (Grass Technologies, West Warwick, RI, USA), delivered via platinum electrodes on both sides of the muscles. The optimum stimulating voltage and optimal muscle length (*L_0_*) were set and muscles were exposed to 10 contractions of 400 ms each at 100 Hz, 30 s apart. One hundred and fifty ms after the initiation of each contraction, muscles were stretched by 9% of *L_0_* over a period of 100 ms at a speed of 0.9 *L_0_*/s and maintained at that level for another 100 ms before returning to the original length. Force loss during the eccentric contraction procedure as well as recovery after 20 min of rest were expressed for every muscle. Test compounds or vehicle were added to the bath 20 min before initiation of the contraction protocol.

In experiments designed to assess membrane permeability, Procion orange (0.2%, w/v) was added to the bath 5 min before the eccentric contraction procedure. After performing the full protocol described above, muscles were briefly washed twice in Ringer solution, blotted, quickly embedded in 5% Tragacanth gum and snap frozen in isopentane cooled in liquid nitrogen. Twenty µm thick sections were cut around the mid-belly of each muscle, fixed in acetone at −20°C, and incubated with wheat germ agglutinin conjugated to AlexaFluor 488 (WGA-AF_488_, Invitrogen), 1 µg/ml in PBS for 1 hour as described to label the extracellular matrix [33]. The amount of Procion orange positive fibers was expressed as a percentage of the total number of fibers, determined from the WGA-AF_488_ counterstain.

### Data presentation and statistical analyses

Results are reported as mean ± S.E.M. Statistical differences between groups were assessed by 1-way ANOVA followed by Fisher LSD multiple comparison post-tests using the GraphPad Prism software, version 6. Differences were considered significant at values of *P*≤0.05. For consistency, all the graphs show the untreated dystrophic and wild-type values as black and light grey columns, respectively. Values obtained with diapocynin, apocynin and blockers of specific pathways appear in green, red and blue, respectively.

## Results

### Diapocynin but not apocynin inhibits ROS production in dystrophic myotubes

**Figure 1:**
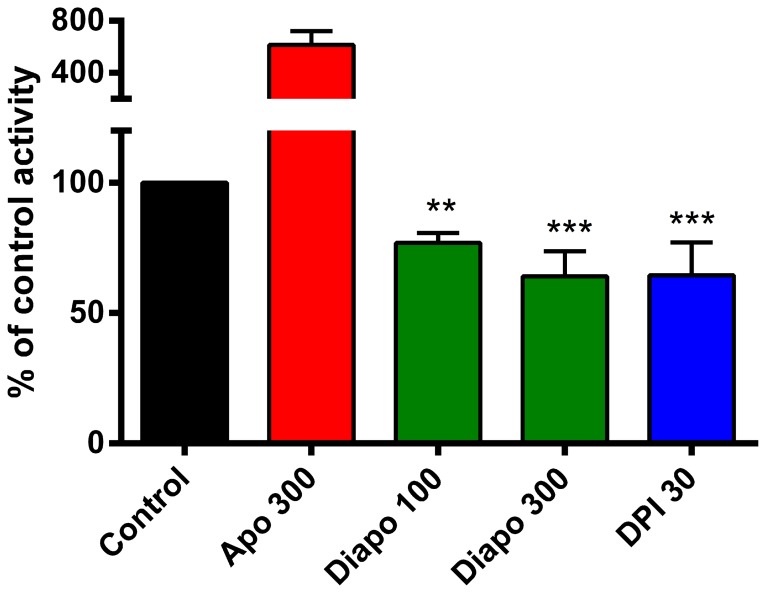
Diapocynin inhibits ROS production in dystrophic myotubes. ROS production in cultured dystrophic myotubes was monitored using DCFH-DA. Fluorescence increments over a period of 20 minutes were quantified in the presence of vehicle or test compounds. Diapocynin (Diapo) caused a concentration-dependent inhibition of ROS production amounting to about 40% at 300 µM, whereas apocynin (Apo) led to a 6-fold increase of fluorescence. The broad flavo-enzyme inhibitor, DPI, commonly used as NOX inhibitor, caused a similar inhibition as 300 µM diapocynin. Concentrations shown on the graph are in µM. ** *P*≤0.01, *** *P*≤0.001 compared to untreated mdx control (n = 3−7).

Treating EDL-MDX-2 myotubes with 100 or 300 µM of diapocynin resulted in a reduction of the total ROS produced. Diapocynin at 300 µM reduced ROS by 36.9±9.6%, a value that was indistinguishable from that of DPI, a potent non-selective NOX inhibitor (Figure 1). By contrast, apocynin caused a 6-fold increase of ROS production.

### Diapocynin but not DPI potently inhibits iPLA_2_ in dystrophic myotubes

**Figure 2:**
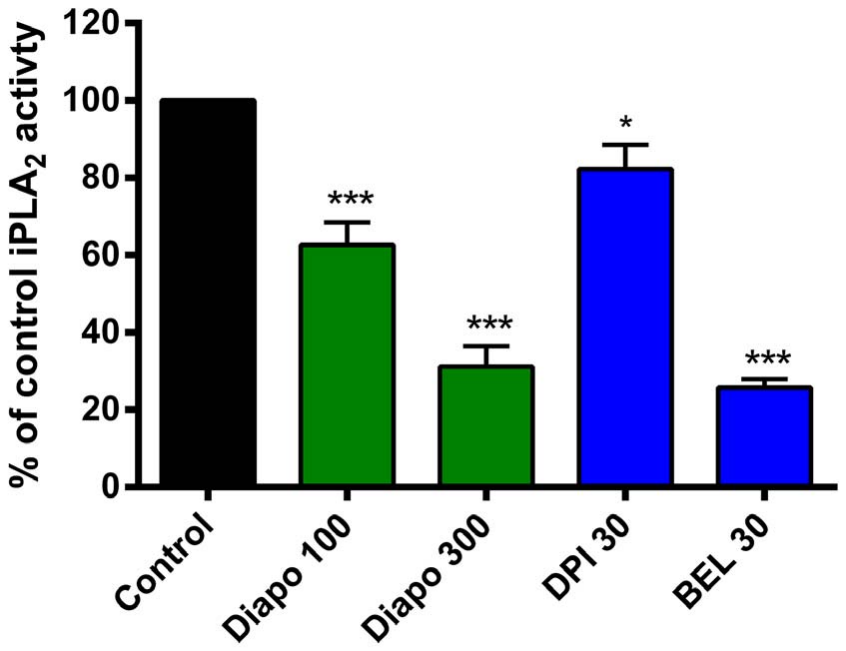
Diapocynin displays potent inhibition of iPLA_2_ in dystrophic myotubes. PED-6 was used as a probe to monitor iPLA_2_ activity in myotubes. Cellular phospholipases cleave this probe to release the fluorescent BODIPY; the rate of formation of this moiety was monitored for 20 minutes. Experiments were performed in the absence of extracellular Ca^2+^ in order to facilitate the activity of iPLA_2_ over other phospholipase isoforms. Diapocynin (Diapo) potently inhibited the iPLA_2_ signal to levels similar to those of the suicide inhibitor, BEL. Note the absence of significant inhibition with DPI. Concentrations shown on the graph are in µM. * *P*≤0.05, *** *P*≤0.001 compared to untreated mdx control (n = 3−8).

In order to minimize the contribution of Ca^2+^-dependent isoforms of PLA_2_, measurements were made on EDL-MDX-2 myotubes in a Ca^2+^-free buffer (PSS−). Diapocynin treatment resulted in an inhibition of the PED-6 signal, amounting to 31.2±5.25% of the control values at 100 µM (Figure 2). At 300 µM, the inhibition reached a level of about 75%, similar to that obtained by 30 µM BEL, a specific iPLA_2_ inhibitor. Interestingly, DPI failed to show a similarly extensive inhibition of PLA_2_ (Figure 2).

### Diapocynin affects ^45^Ca^2+^ influx through SAC and SOC

**Figure 3:**
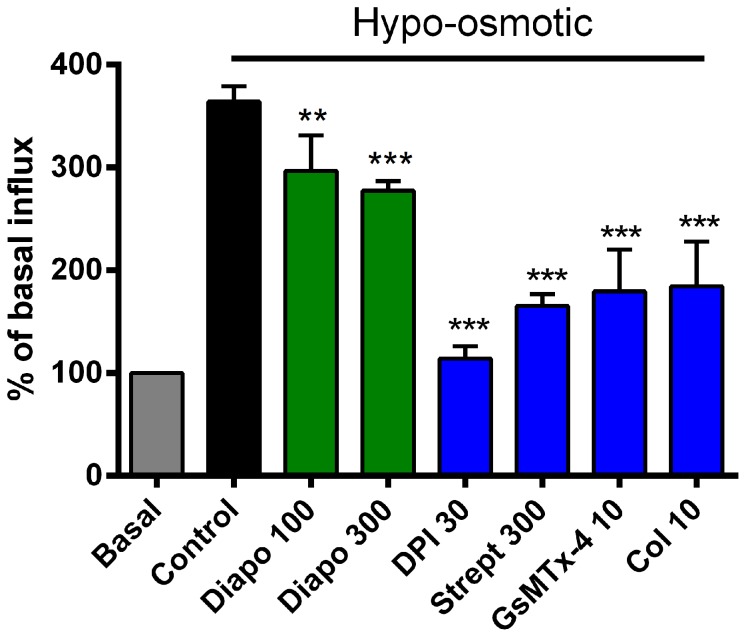
Modulation of hypo-osmotic shock induced Ca^2+^-influx in dystrophic myotubes. Exposing myotubes to a hypotonic PSS containing 1 µCi ^45^Ca^2+^ induced a 3.7-fold increase in ^45^Ca^2+^-influx compared to isotonic PSS. Diapocynin (Diapo) treatment resulted in a 30% inhibition of the stimulated influx, whereas DPI caused an inhibition to control levels. The SAC blockers, streptomycin (Strept) and Grammatoxin (GsMTx-4), or the microtubule disruptor, colchicine (Col), caused a similar inhibition of about 70%. Concentrations shown on the graph are in µM. ** *P*≤0.01, *** *P*≤0.001 compared to untreated mdx control (n = 4−7).

Due to the central role of Ca^2+^ influx in the pathology in DMD, we evaluated the effect of diapocynin on Ca^2+^ influx through SAC and SOC. Treating the myotubes with 100 or 300 µM of diapocynin resulted in a small but significant inhibition of SAC influx with a value of 25.6±12.9% and 32.8±3.6% of control values, respectively (Figure 3). DPI, however, led to an almost complete inhibition of the influx. The classical SAC inhibitors, streptomycin and grammatoxin (GsMTx-4, a peptide isolated from the venom of the tarantula spider *Grammostola spatulata*), as well as the microtubule disruptor, colchicine, inhibited about 70% of the SAC influx (Figure 3).

**Figure 4:**
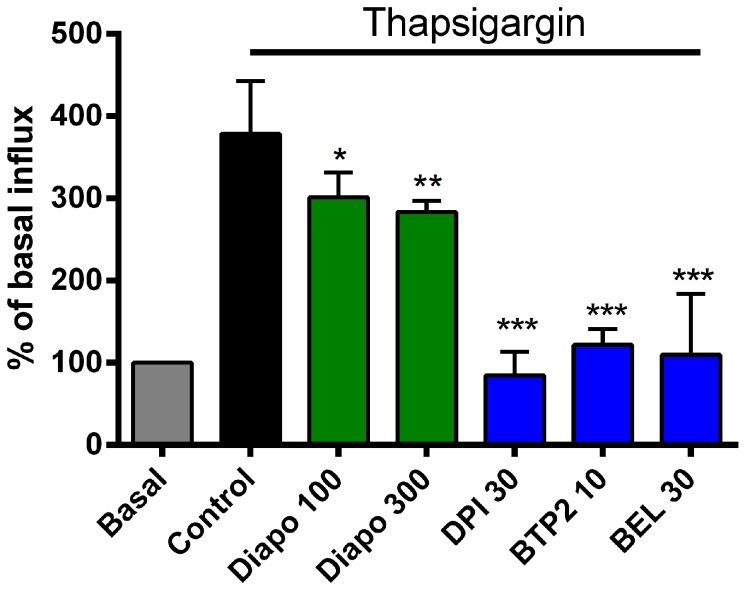
Modulation of Ca^2+^ influx in dystrophic myotubes induced by store-depletion. Thapsigargin (5 µM) treatment was used to deplete the sarcoplasmic Ca^2+^ stores leading to activation of SOC influx. Re-addition of ^45^Ca^2+^-containing buffer resulted in an almost 4-fold increase in ^45^Ca^2+^-influx compared to non-treated cells. Diapocynin (Diapo) treatment had a small effect, whereas DPI showed a complete inhibition, similar to the one observed with BTP2, a commonly used SOC blocker, or BEL. Concentrations shown on the graph are in µM. * *P*≤0.05, ** *P*≤0.01, *** *P*≤0.001 compared to untreated mdx control (n = 3−10).

When SOC influx was studied using thapsigargin, similar patterns of inhibition were observed with the test compounds. Diapocynin at 300 µM inhibited about 34% of the induced influx whereas DPI inhibited it to a level of un-stimulated cells (Figure 4). Similarly, a reference SOC blocker, BTP2, and the iPLA_2_ inhibitor, BEL, efficaciously blocked the induced influx down to basal levels (Figure 4).

### Diapocynin prevents eccentric contraction-induced damage

**Figure 5:**
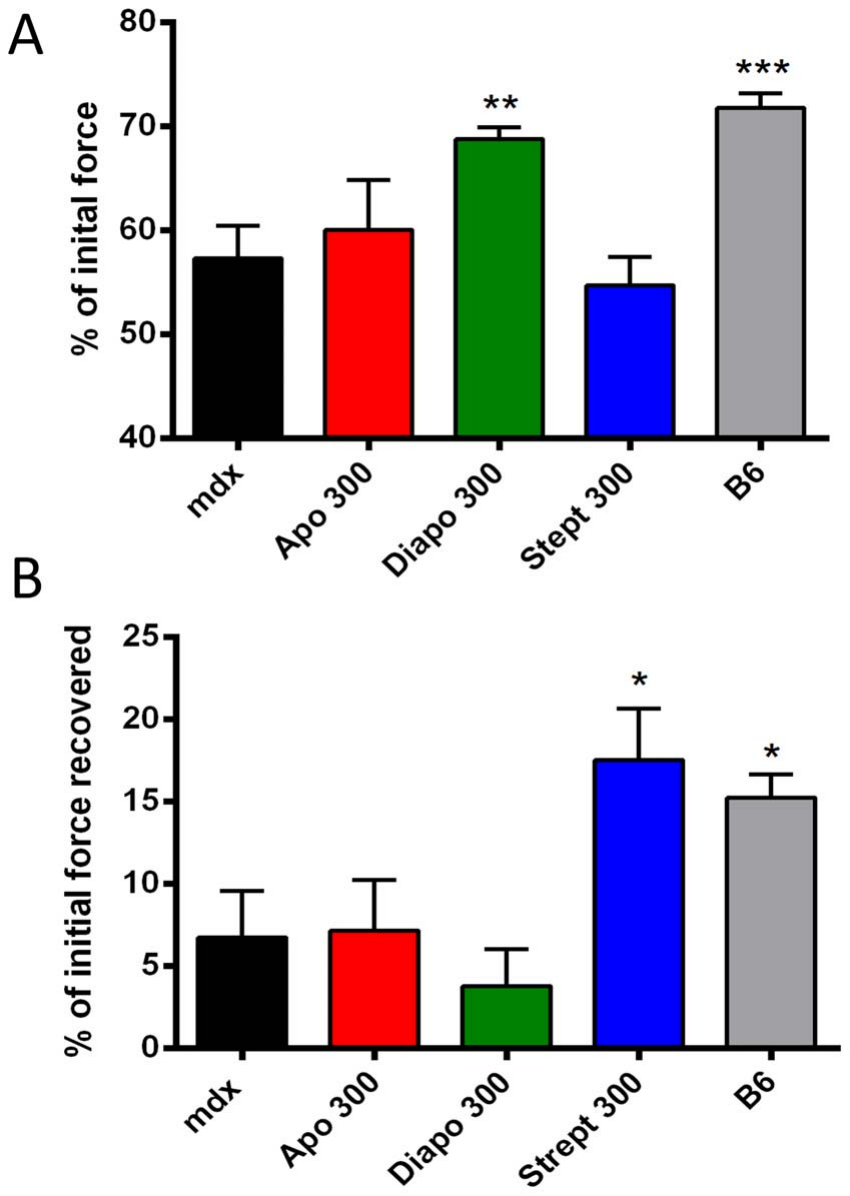
Diapocynin abrogates force loss in eccentrically contracted EDL muscles whereas streptomycin promotes the recovery of force loss after a period of rest. EDL muscles from wild-type (B6) and dystrophic mice were exposed to 10 eccentric contractions at 100 Hz lasting 400 ms during which they were stretched to a value of 109% of their optimal length. (A) Remaining force after 10 eccentric contractions. Note the increased force loss in dystrophic muscle (mdx) compared to wild type (B6) muscle. Of the tested compounds, only diapocynin (Diapo) abrogated the force loss seen in this assay while apocynin (Apo) failed to show such an effect. (B) Force recovered after 20 minutes of rest. Streptomycin (Strept) caused a marked recovery exceeding the one of wild-type muscles. Concentrations shown on the graph are in µM. * *P*≤0.05, ** *P*≤0.01 compared to untreated mdx control (n = 4−9).

Exposing dystrophic EDL muscles to 10 repeated eccentric contractions resulted in a greater force loss compared to their wild-type counterparts. Incubation of the muscles with 300 µM of diapocynin prior to the contractions prevented force loss to near wild-type levels (Figure 5A). However, such a protective effect was not observed in the groups treated with streptomycin, as judged immediately after the last eccentric contraction.

Then muscles were allowed to recover from the damaging protocol for 20 minutes and the force was measured subsequently. Again, wild-type muscles recovered almost two times better than dystrophic ones: the force recovered was about 15% of pre-exercise values (Figure 5B). Streptomycin-treated muscles displayed a striking recovery compared to dystrophic and wild-type controls while it failed to prevent force loss during the active phase of the assay (Figure 5B).

**Figure 6:**
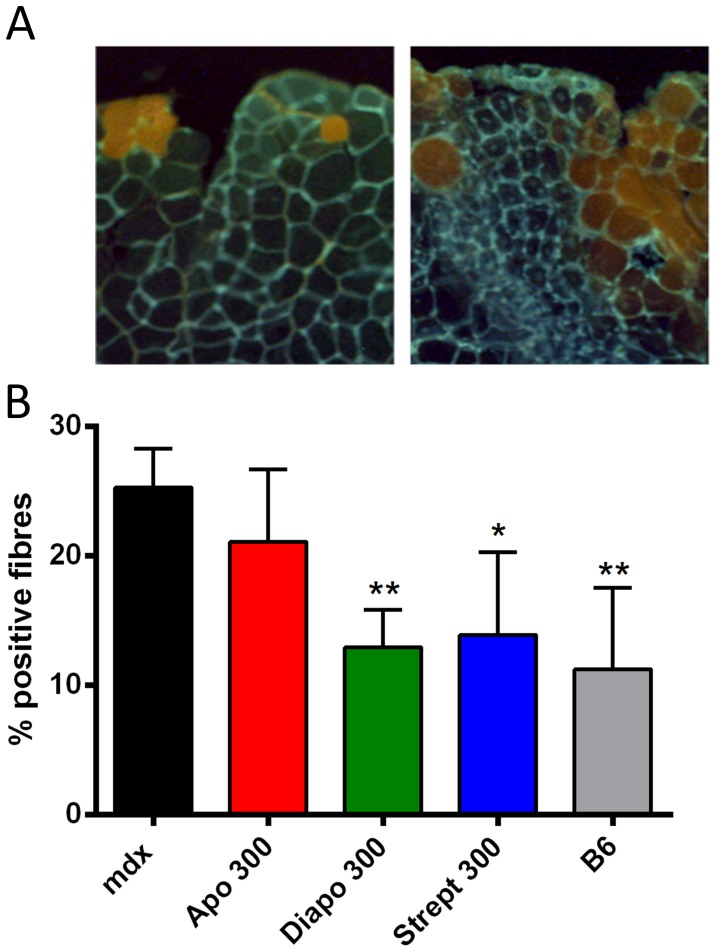
Protection of sarcolemmal integrity by diapocynin in eccentrically contracted EDL muscles. Procion orange is a membrane-impermeable dye that enters only cells with damaged membranes. In this assay, EDL muscles were exposed to 10 eccentric contractions in physiological buffer containing 0.2% Procion orange. Muscles were subsequently rinsed twice in physiological buffer and embedded in tragacanth gum. Twenty micrometer thick sections were made around the mid-belly region of the muscles and the percentage of Procion orange-positive fibers were quantified. Representative section of wild type (A, left) and dystrophic (A, right) EDL muscles exposed to eccentric contractions in the presence of Procion orange. (B) Quantification of Procion orange-positive fibers in the experimental groups. Dystrophic muscle (mdx) displayed a 2-fold increase in dye uptake as compared to wild-type muscle (B6). Diapocynin (Diapo) or streptomycin (Strept) treatment protected the muscle from increased membrane damage, thus lowering the values of the stained fibers down to those of the non-dystrophic controls. Apocynin (Apo) did not offer a protection in this assay. Concentrations shown on the graph are in µM. * *P*≤0.05, ** *P*≤0.01 compared to untreated mdx control (n = 4−8).

To investigate sarcolemmal integrity after damaging contractions, experiments were performed in the presence of the vital dye Procion orange, which was equilibrated into the buffer 5 min before the initiation of the contraction protocol and kept till the end of the recovery phase. Exposing dystrophic EDL muscles to 10 eccentric contractions resulted in a 2-fold increased dye uptake compared to wild-type muscles (Figure 6). Diapocynin reduced this uptake to the value of non-dystrophic muscle, whereas apocynin had no significant effect. The SAC blocker, streptomycin, also had a protective effect, probably by rendering the sarcolemma more resilient to stretch-induced damage (Figure 6).

## Discussion

Our study demonstrates the ability of diapocynin, but not of apocynin, to inhibit ROS production in skeletal muscle cells. We also show that diapocynin inhibits iPLA_2_, reduces Ca^2+^ influx through SOC and SAC and protects muscle from eccentric contraction-induced force loss.

There is a lack of selective, non-toxic NOX inhibitors, the best known being apocynin. It was first isolated by Schmiedeberg in 1883 from the roots of *Apocynum cannabinum*, but it was only in the 1990’s that apocynin was found to inhibit NOX-dependent ROS production (for a review see [34]). Apocynin’s ability to reduce NOX-mediated ROS generation results from both altered translocation of regulatory subunits to the membranes and prevention of their proper assembly with the transmembrane core protein gp91^ph^°^x^ [35]. This inhibition was observed only in activated phagocytic cells but was completely lacking in other cell types and even had a ROS-promoting effect in non-phagocytic cells [24]. In attempts to explain these findings, apocynin was proposed to act as a pro-drug undergoing two different metabolic pathways, namely oxidative dimerization by myeloperoxidases in activated phagocytic cells to form diapocynin, which is thought to be the active moiety inhibiting the enzyme [26], [36], or generation of a transient pro-oxidant apocynin radical that can subsequently oxidize sensitive sulfhydryl groups of NOXes [37]. Diapocynin was found to be superior to apocynin in inhibiting not only NOX activity acutely [36], but also gp91^ph^°^x^ expression, TNF-α and IL-10 production in response to LPS challenge in non-phagocytic cells following a 24 hours incubation [25].

Diapocynin inhibited ROS production in skeletal muscle myotubes whereas apocynin showed a 6-fold increase in ROS output (Figure 1). This finding is in accordance with a report on non-phagocytic cells [24]. The pro-oxidant activity of apocynin depends on its prior oxidation to transient free radicals, such as apocynin radicals [37]. Such radicals have been reported to cause a 7-fold increase in glutathione oxidation and an even 100-fold increase in NADPH oxidation [38]. These results reinforce previous reports that diapocynin is the active species inhibiting NOX and that apocynin primarily serves as pro-drug of its oxidized dimer [36].

Both diapocynin and DPI inhibited ROS production in our cellular model to a similar extent (Figure 1). DPI is not a selective NOX inhibitor but a wide-spectrum flavo-enzyme inhibitor causing also inhibition of CytP450, nitric oxide synthases and xanthine oxidase [39]. The similar extent of inhibition of ROS production exhibited by DPI and diapocynin in myotube cultures (about 40%) is consistent with the fact that NOXes are major ROS contributors in skeletal muscle tissue [17], [18]. A docking model for apocynin and some of its analogues into the complex p67^ph^°^x^-p47^ph^°^x^ was recently proposed and it was shown that diapocynin had the highest affinity score of all tested compounds [40]. This supports earlier reports that diapocynin and apocynin might have the same inhibitory mechanism on NOX, namely binding to p47^ph^°^x^, thus preventing the assembly of the subunits required for NOX2 activity [36]. Whether diapocynin inhibits NOX4, which does not require translocation of subunits, remains to be investigated.

We have shown previously the involvement of iPLA_2_ in modulating Ca^2+^ entry into dystrophic myotubes and fibers and that pharmacological inhibition of iPLA_2_ blocks the enhanced Ca^2+^ entry [6], [31]. Diapocynin treatment fully inhibited iPLA_2_ in our dystrophic myotubes to levels indistinguishable from those elicited by the suicide iPLA_2_ inhibitor BEL (Figure 2). Likely targets of diapocynin could be NOXes, located in close proximity of the sarcolemma. Their inhibition would lead not only to decreased superoxide anion radical (O_2_^•−^) formation, but also to a lower production of lipid peroxides. Such peroxides are known to be superior substrates for iPLA_2_ compared to native phospholipids [41]. Alternatively, a direct inhibition of iPLA_2_ by diapocynin cannot be ruled out. One important consideration in the effect of diapocynin on its targets is lipophilicity. Diapocynin is 13 times more lipophilic than apocynin [42], enabling it to cross membranes freely. Such a characteristic can lead to membrane accumulation, which could bring the compound into close vicinity of its targets. By contrast, the lack of potent iPLA_2_ inhibition by DPI might be attributed to its reduced ability to accumulate in biological membranes and therefore the lack of potent inhibitory action in this specific cellular compartment. Also, DPI is known to be a “dirty” compound, therefore, non-specific actions of DPI on other targets may ultimately mitigate the cellular response [43], [44].

Diapocynin inhibited calcium influx through SAC to a lesser extent than the classical inhibitors, streptomycin and GsMTx-4 (Figure 3). In an elegant series of papers, Lederer, Ward and colleagues showed that membrane stretch activates NOXes to produce ROS, which subsequently activate SAC [28], [45]. Diapocynin, through its ability to inhibit NOX2, would lead to the disruption of this cascade and eventually block cation influx through SAC. The same authors also proposed the microtubular network to convey the mechanical stretch to NOX and that colchicine, a microtubule disruptor, leads to the inhibition of SAC influx in dystrophic FDB fibres [28]. Our present results confirm these findings in dystrophic myotubes, as colchicine inhibited SAC influx to the same extent as streptomycin or GsMTx-4 (Figure 3).

SOC influx using thapsigargin as a trigger revealed that diapocynin partially inhibited this influx but this inhibition became significant only at 300 µM (Figure 4). This can be attributed to iPLA_2_ inhibition, as evidenced by the inhibition of the influx by BEL and is in line with our previous report [46]. Of note, BEL does not only inhibit SOC as an iPLA_2_ inhibitor, but it also inhibits directly several TRP channels, an effect that might contribute to its full blockade seen here, as well as previously, in our hands [47]. This can explain why BEL appeared to be more efficacious than diapocynin in preventing SOC-mediated Ca^2+^ influx in our hands.

DPI caused full inhibition of both SAC and SOC, exceeding the levels reached by the most selective inhibitors of the targeted channel types, namely GsMTx-4 and BTP2, respectively (Figures 3 and 4). This cannot be explained by the sole ability of DPI to inhibit flavo-enzymes, such as NOXes. In earlier work, in which patch-clamp techniques were used on isolated pulmonary smooth muscles, it was reported that DPI inhibited both Ca^2+^ and K^+^ channels at concentrations of 3 and 10 µM, independent of its NOX modulating activity [43]. Since the concentrations used in this study are 3–10 times higher than those reported to have channel modulating activity, such an inhibition of channels might well play a role. To the best of our knowledge, this is the first report showing that DPI has such a potent inhibitory effect on Ca^2+^-influx stimulated by membrane stretch and by internal store emptying. However, considering the effect of DPI on SAC and SOC influx, and its broad inhibitory profile on flavo-enzymes, we suggest that DPI should not be used as an experimental tool for blocking NOXes in a similar context.

**Figure 7:**
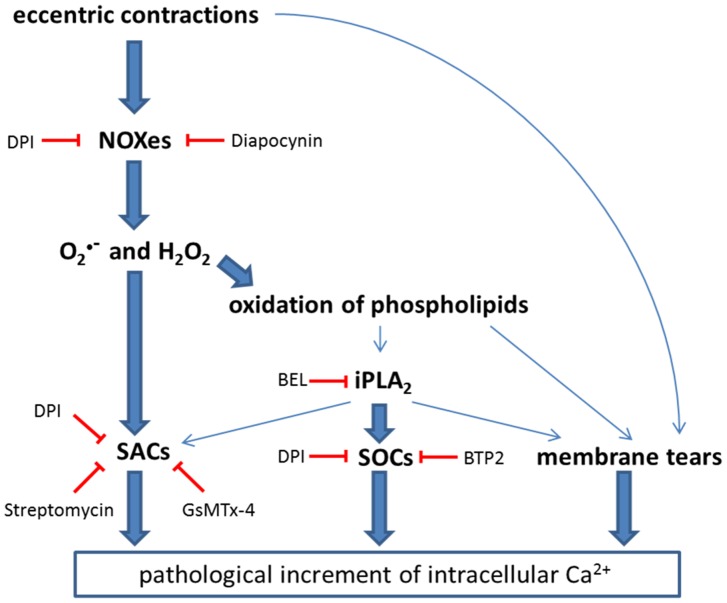
Proposal for the cascade implicated in the force loss in eccentrically contracting dystrophic muscles. The illustration shows a possible cascade of the events taking place in eccentrically contracting dystrophic muscle. Thick arrows highlight pathways reported to play a major role in muscle function, namely NOX-SACs, iPLA_2_-SOCs and membrane tears. Thin arrows point to suggested links between the main pathways involved. The blunted red arrows show the inhibitory effect of compounds used in the current study. The suggested cascade results in an elevation of intracellular Ca^2+^, an event known to activate multiple downstream pathways playing a role in the dystrophic pathology.

Exposing isolated EDL muscles to 10 repeated eccentric contractions resulted in an increased force loss of dystrophic muscle compared to its wild-type counterpart. This has been first reported 20 years ago by Petrof *et al.*, who also showed enhanced membrane disruption and dye uptake as a consequence of such contractions [48]. Numerous attempts were carried out to investigate the mechanisms causing this force loss, which led to the notion that the two major determinants involved are disruption of Ca^2+^ homeostasis and myofibrillar disorganization [49]. In line with an inhibition of increased Ca^2+^ influx, SAC blockers, such as streptomycin or GsMTx-4, or removal of extracellular Ca^2+^ have been shown to be beneficial in promoting force recovery following eccentric contractions [27], [50]. Recently, ROS came into play as mediators carrying an essentially cytotoxic message, as evidenced by increased resistance to eccentric damage in mdx mice by *N*-acetylcysteine treatment or by transgenic overexpression of catalase [10], [51]. A recent study has also shown that DPI decreased force loss induced by eccentric contractions in isolated FDB fibers [20]. In the current study, we show that diapocynin was the only compound investigated that abolished force loss occurring during eccentric contractions, whereas streptomycin failed to do so (Figure 5). However, when measured after 20 minutes of recovery, streptomycin-treated muscle recovered to an extent similar to the one of diapocynin. These results support earlier findings that both SAC and ROS contribute to the force loss induced by eccentric contractions in dystrophic muscles; however, their specific roles in the different stages of force loss and recovery might be different. In support to this, it has been convincingly proposed recently that ROS production by NOXes precedes SAC activation seen in stretching conditions [28], [45]. This can explain our findings that diapocynin prevented force loss whereas streptomycin promoted the recovery after the contractions. Gervasio *et al.* showed that increased levels of pro-oxidants, such as H_2_O_2_, lead to autophosphorylation of Src kinase and subsequent activation of SAC and that the anti-oxidant tiron or the Src kinase inhibitor PP2 inhibited increased Ca^2+^ influx after eccentric contractions [52]. Another study showed that addition of SAC blockers just after eccentric contractions was sufficient to obtain a protective effect [50]. Taken together, these findings point towards increased ROS production in eccentrically contracting muscle having a dual role, the first one promoting force loss by increasing ROS above the levels that are required for optimal force production [53], and the second one activating Ca^2+^ influx through SAC, promoting membrane damage and finally enhancing dye uptake. NOX inhibitors would prevent the first step of the cascade while SAC blockers would inhibit the final step. This is illustrated in Figure 7. Our results with Procion orange dye uptake re-enforces these finding and shows that both diapocynin, and to a lesser extent streptomycin, protected the muscles form membrane damage induced by stretching contractions.

ROS contribute to normal cellular homeostasis and fine tuning of metabolic processes [54]. Many anti-oxidants tested so far in dystrophic mice are global ROS scavengers that do not discriminate between sources of ROS. Their ability to alter the cells’ redox status instead of targeting a specific source of ROS might explain why such anti-oxidant therapies showed only limited improvement of the dystrophic condition. Targeting overactive NOXes with diapocynin might confer higher experimental and therapeutic potential compared to global anti-oxidants. In additions, a recent study showed that diapocynin has a good pharmacokinetic profile when administered orally and that such a treatment resulted in a neuroprotective effect in models of Parkinson’s disease [55]. Another study revealed that diapocynin has a powerful anti-inflammatory activity independent of its ROS modulating capability [56]. Another consideration is the possibility that diapocynin act as a direct anti-oxidant through ROS scavenging thanks to the presence of 2 phenolic groups [57]–[59]. Thus diapocynin would have a dual mechanism of action, both contributing to ROS reduction.

Altogether, our results and data by others suggest that diapocynin is a promising compound with potential for treating dystrophic muscle diseases and is worthy of further evaluation. Towards this goal we currently are performing an *in vivo* investigation of diapocynin in mdx mice.
